# Supplementary material for: Interpretable Antibody–Antigen Structural Interface Prediction via Adaptive Graph Learning and Cyclic Transfer
Source: bioRxiv. 2026 Apr 12:2026.04.09.717547. Preprint. [Version 1] doi: 10.64898/2026.04.09.717547 (PMC13081910; doi:10.64898/2026.04.09.717547)
Supplement: Supplement 1 [file media-1.pdf]

Supplementary material

Table S1 Performance of models with various structure on three datasets

Dataset	MSA layers	GNN layers	Attention layers	Antibody AUPR	Antibody AUC-ROC	Antigen AUPR	Antigen AUC-ROC
MIPE	1	4	2	0.725	0.981	0.247	0.753
	1	6	2	0.732	0.981	0.256	0.758
	1	6	3	0.742	0.981	0.282	0.775
	1	6	4	0.726	0.981	0.222	0.757
	1	8	2	0.729	0.981	0.244	0.759
	1	8	3	0.722	0.981	0.246	0.761
	1	8	4	0.726	0.981	0.223	0.757
	1	10	5	0.726	0.981	0.215	0.747
paragraph	0	10	5	0.747	0.982	0.449	0.801
	1	0	5	0.584	0.964	0.409	0.766
	1	10	0	0.726	0.980	0.454	0.798
	1	10	5	0.751	0.982	0.472	0.812
	1	2	1	0.721	0.980	0.461	0.810
	1	4	2	0.735	0.981	0.458	0.806
	1	6	3	0.747	0.982	0.461	0.809
	1	8	4	0.746	0.982	0.458	0.803
	1	10	4	0.752	0.982	0.469	0.810
	1	10	6	0.753	0.982	0.464	0.811
	1	12	4	0.752	0.982	0.452	0.798
	1	12	5	0.752	0.982	0.458	0.801
	1	12	6	0.753	0.982	0.461	0.805
	1	14	7	0.753	0.982	0.452	0.799
VASCO	1	10	5	0.716	0.795	0.528	0.943
	1	12	6	0.730	0.964	0.541	0.932
	1	14	7	0.700	0.963	0.498	0.936
	1	16	8	0.693	0.963	0.464	0.931
